# Supplementary material for: Downregulation of the GhROD1 Gene Improves Cotton Fiber Fineness by Decreasing Acyl Pool Saturation, Stimulating Small Heat Shock Proteins (sHSPs), and Reducing H2O2 Production
Source: Int J Mol Sci. 2024 Oct 19;25(20):11242. doi: 10.3390/ijms252011242 (PMC11509027; doi:10.3390/ijms252011242)
Supplement: Supplementary file 1 [file ijms-25-11242-s001.zip › Supplementary materials - Figures-revision Round 3.pdf]

Supplementary materials: Figures

Downregulation of the *GhROD1* gene Improves Cotton Fiber Fineness by Decreasing Acyl Pool Saturation, Stimulating Small Heat Shock Proteins (sHSPs), and Reducing H_2O_2 Production

Bo Ding [1,2], Bi Liu [1,2], Xi Zhu [1,2], Huiming Zhang [1,2], Rongyu Hu [1,2], Silu Li [1,2], Liuqin Zhang [1,2], Linzhu Jiang [1,2], Yang Yang [1,2], Mi Zhang [1,2], Juan Zhao [1,2], Yan Pei [1,2] and Lei Hou [1,2,*]

1 College of Agronomy and Biotechnology, Southwest University, Chongqing, 400715, P.R. China; 15086655435@163.com (B.D.); morningrain7@sohu.com (B.L.); zhuxi0603@email.swu.edu.cn (X.Z.); zhang15828612304@163.com (H.Z.); 18509175153@163.com (R.H.); lisilu20010817@outlook.com (S.L.); zhangliuqin0715@163.com (L.Z.); linzhuhh@163.com (L.J.); neo5334@163.com (Y.Y.); selenazm@swu.edu.cn (M.Z.); zhaojuan0920@swu.edu.cn (J.Z.); peiyan3@swu.edu.cn (Y.P.)

2 Chongqing Key Laboratory of Application and Safety Control of Genetically Modified Crops, Southwest University, Chongqing 400715, China

* Correspondence: houlei@swu.edu.cn; Tel./Fax: +86-023-68251264

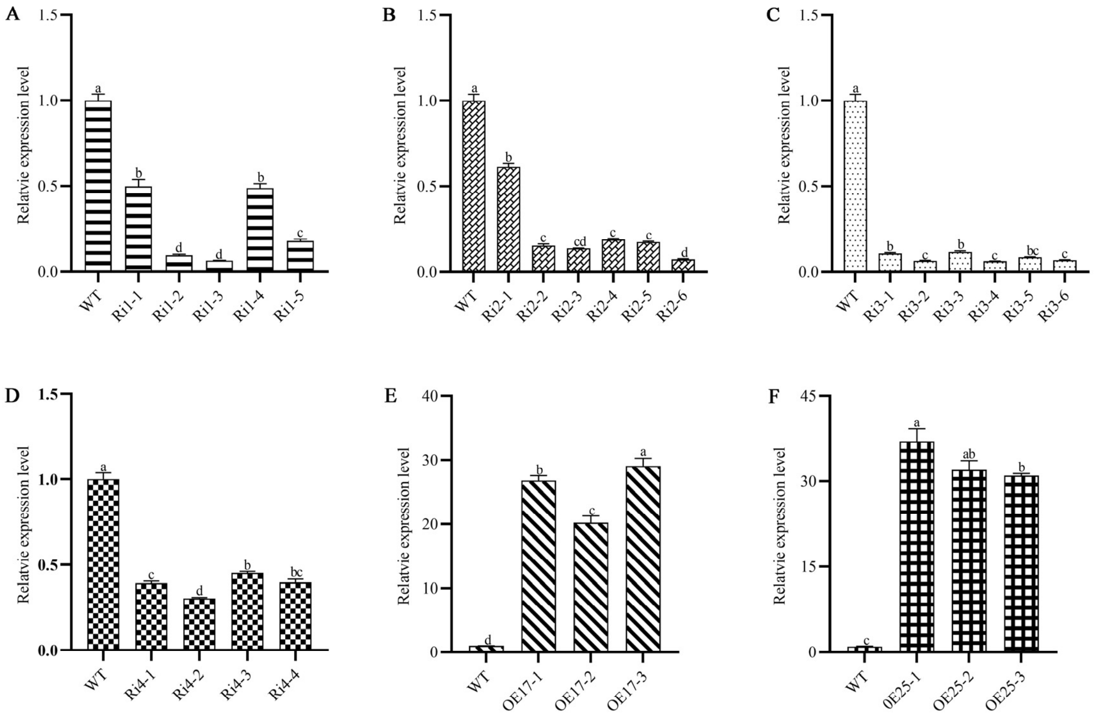

Figure S1: Screening of *GhROD1* transgenic plants. (A-D) Relative expression level of *GhROD1* in transgenic plants. (A) Ri1, (B) Ri2, (C) Ri3, and (D) Ri4. (E and F) Relative expression level of *GhROD1* in transgenic plants. (E) OE17 and (F) OE25. *GhHis3* served as the internal reference gene. Data are presented as mean ± SD (n = 3). Differences between transgenic lines and wild-type were analyzed by one-way ANOVA followed by Tukey's test. Different lowercase letters indicate significant differences among groups.

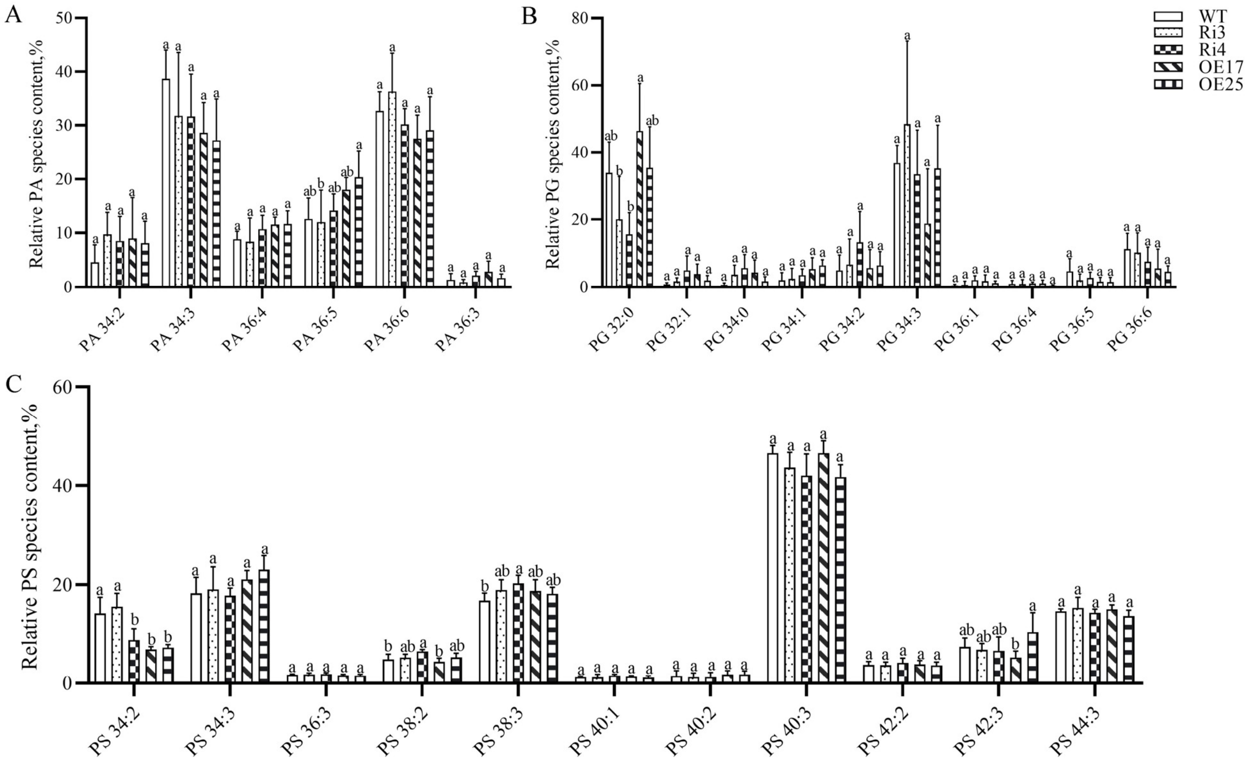

Figure S2: Individual molecular species profiles quantified by ESI-MS/MS for PA, PG, and PS in extracts from transition stage (20 DPA) fibers. (A), (B), (C) PA, PG, PS molecular species profile. WT: wild-type (*Gossypium hirsutum* L. cv. Jimian14). Ri3 and Ri4: *GhROD1*-downregulated lines. OE17 and OE25: *GhROD1*-upregulated lines. PA: phosphatidic acid. PG: phosphatidylglycerol. PS: phosphatidylserine. Numerical designations represent the total number of acyl carbons: total number of double bonds. Data for each molecular species represent the mean and SD of five independent extractions and are presented as mol% of total glycerophospholipids. Differences between transgenic lines and wild-type were analyzed by one-way ANOVA fol-lowed by Tukey's test. Different lowercase letters indicate significant differences among groups. Components with lipid molecular species below 1% are not shown in the figure.

A

B

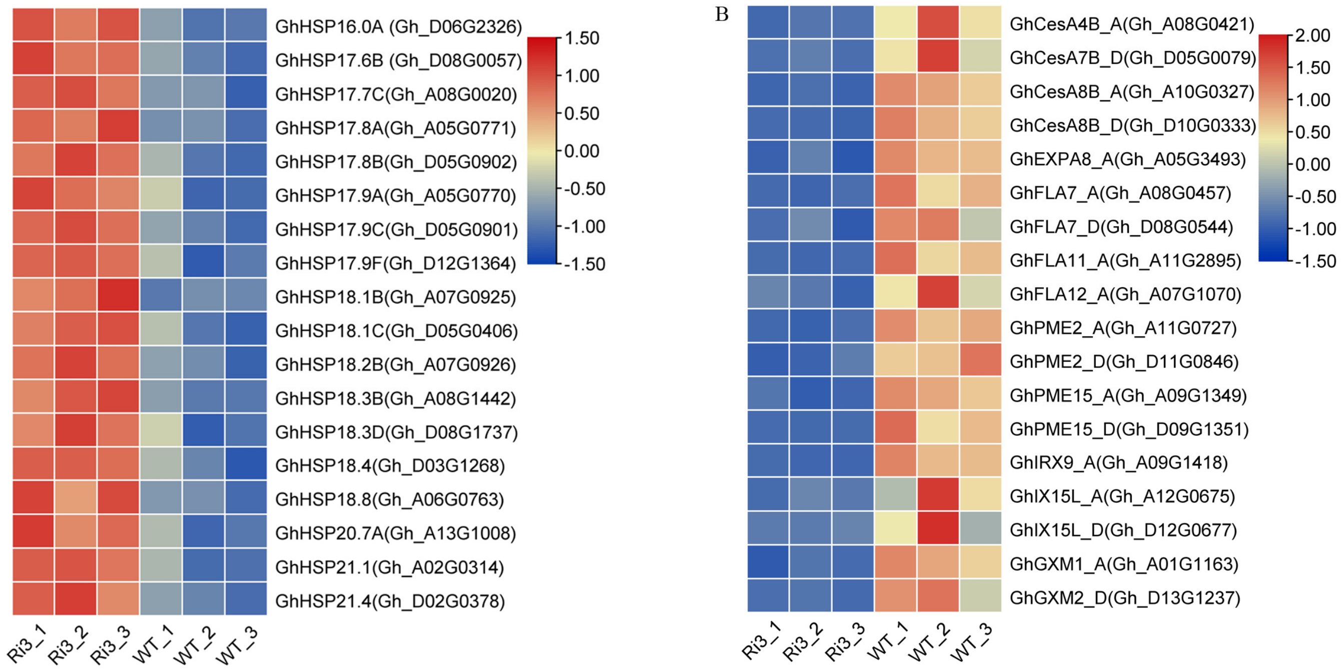

Figure S3: Heatmaps of *sHSPs* and cell wall synthesis-related genes in 16-DPA fibers of Ri3 and wild-type. (A) Heatmap of *sHSPs* expression in Ri3 and wild-type 16-DPA fibers. (B) Heatmap of cell wall synthesis-related genes expression in Ri3 and wild-type 16-DPA fibers. Note: All FPKM values are log2 transformed. Blue indicates relatively lower expression levels, while red indicates relatively higher expression levels.

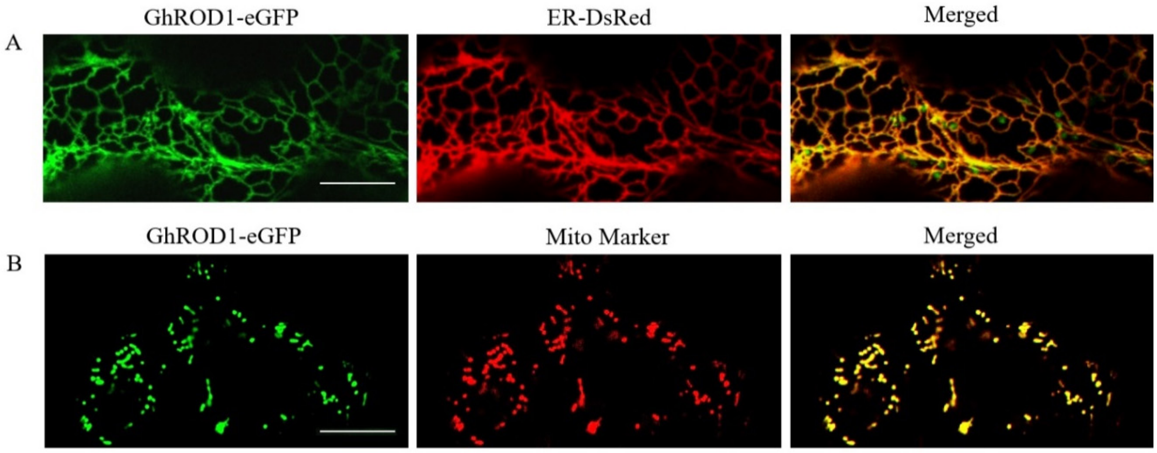

Figure S4: Subcellular localization of GhROD1. (A) GhROD1-eGFP colocalizes with the ER marker protein. The *proGhROD1::GhROD1-eGFP* and *ER-DsRed* constructs were co-expressed in leaves of *Nicotiana benthamiana*. (B) GhROD1-eGFP colocalizes with the mitochondrial marker protein. The *proGhROD1::GhROD1-eGFP* and mitochondrial marker constructs were co-expressed in leaves of *Nicotiana benthamiana*. Scale bar = 10 μm.
