# Supplementary figures and images for: Downregulation of the GhROD1 Gene Improves Cotton Fiber Fineness by Decreasing Acyl Pool Saturation, Stimulating Small Heat Shock Proteins (sHSPs), and Reducing H2O2 Production
Source: Int J Mol Sci. 2024 Oct 19;25(20):11242. doi: 10.3390/ijms252011242 (PMC11509027; doi:10.3390/ijms252011242)

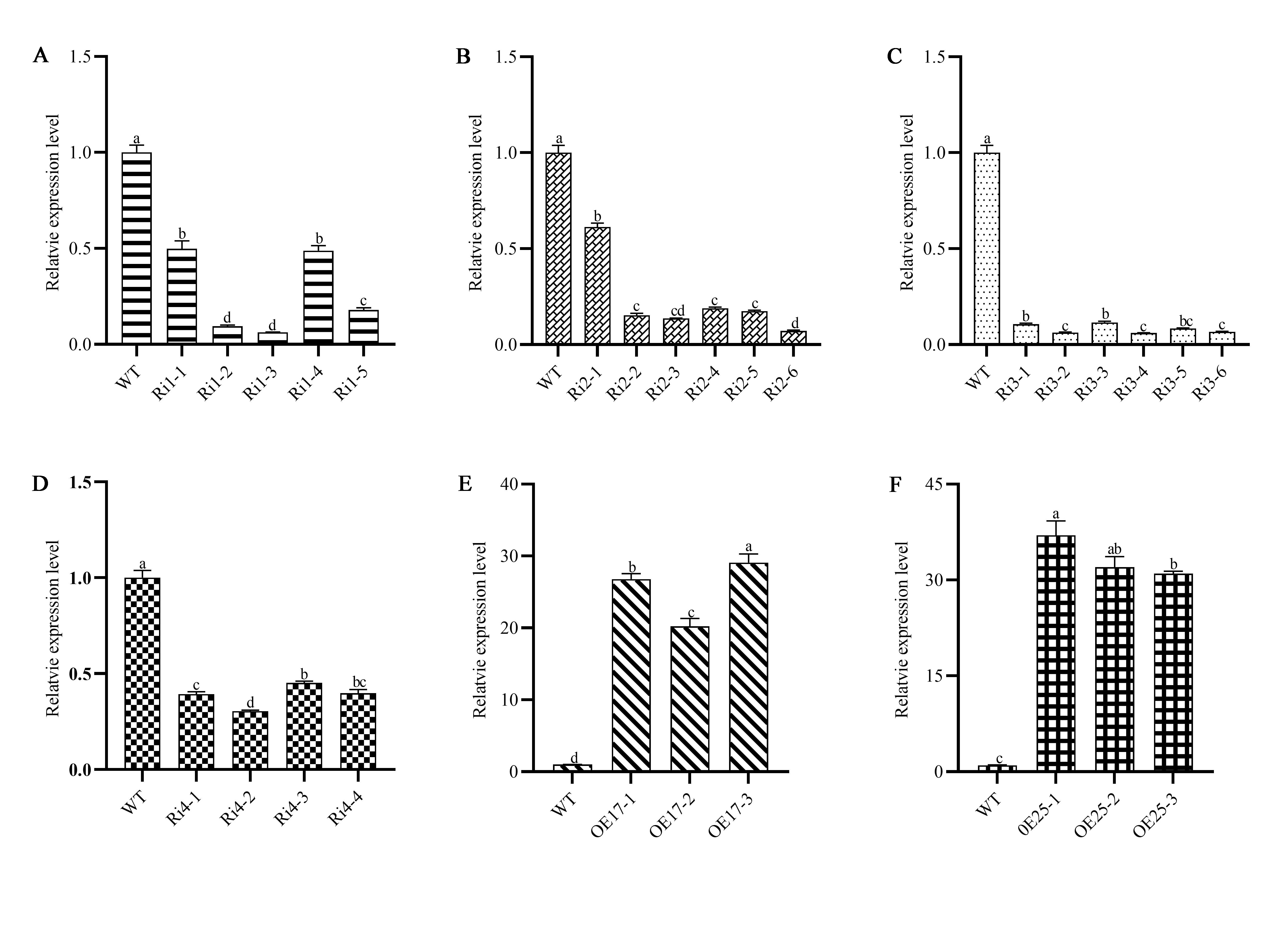

Supplement: Supplementary file 1 [file ijms-25-11242-s001.zip › Figure S1.tif]

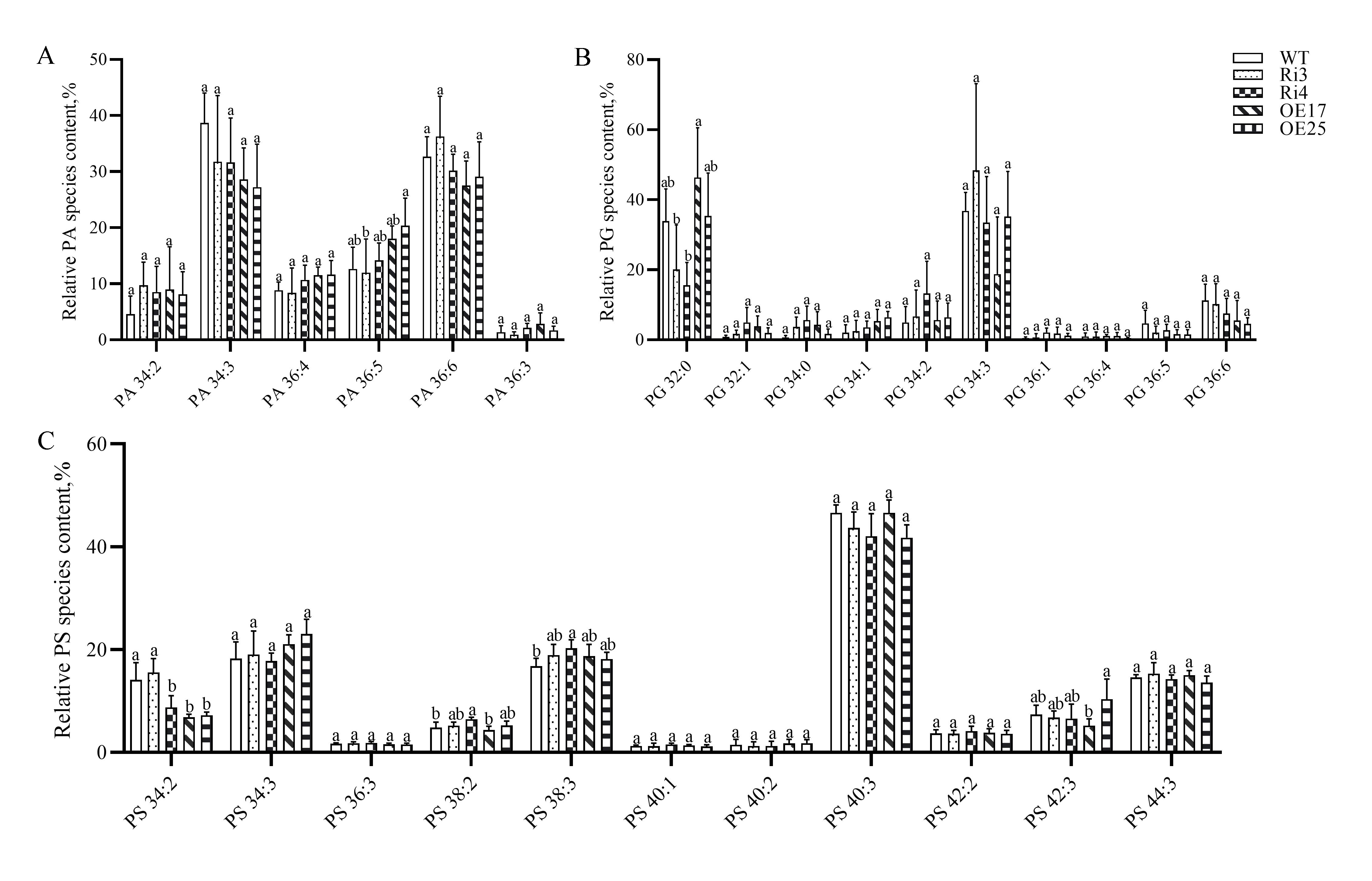

Supplement: Supplementary file 1 [file ijms-25-11242-s001.zip › Figure S2.tif]

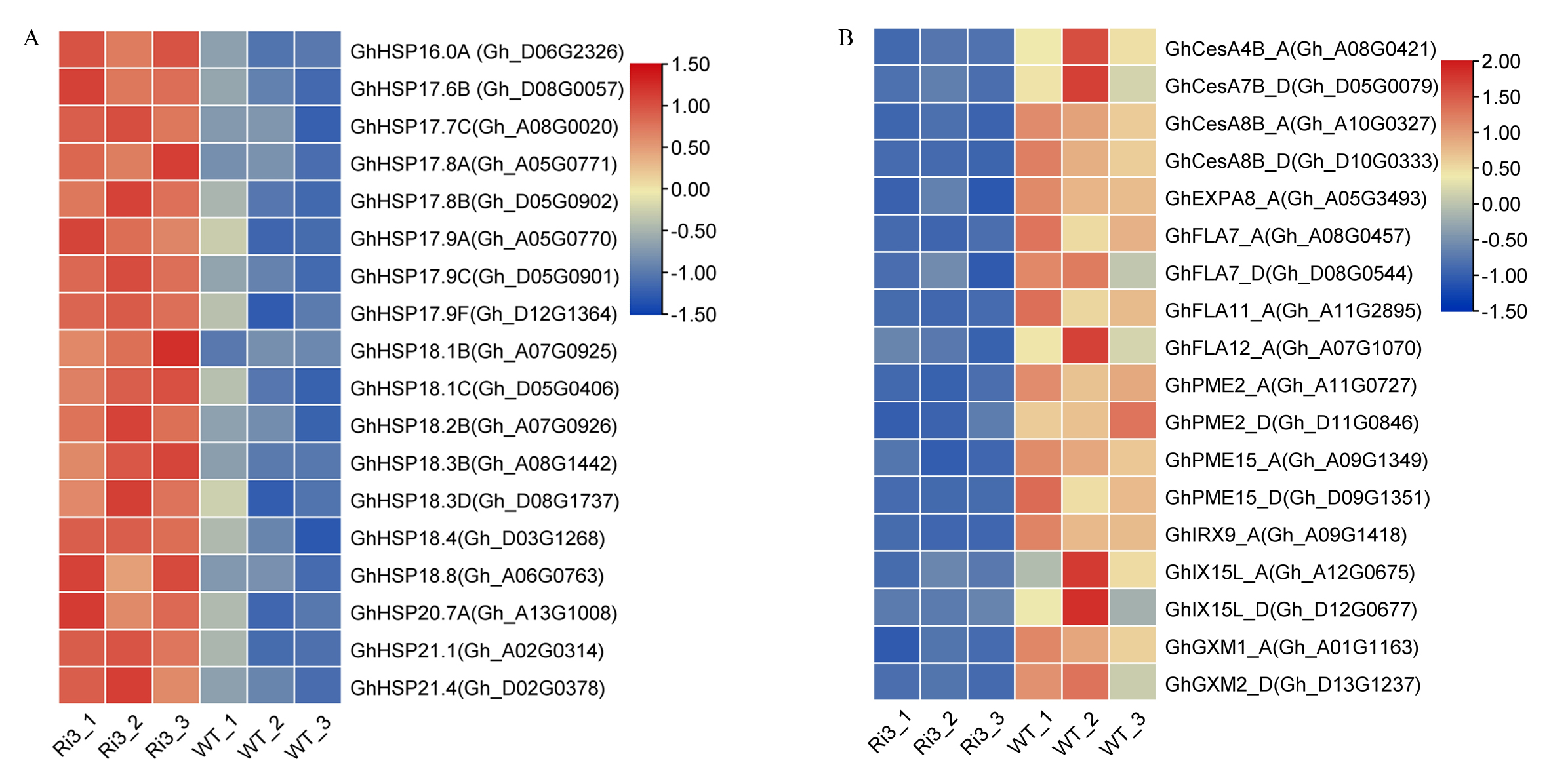

Supplement: Supplementary file 1 [file ijms-25-11242-s001.zip › Figure S3.tif]

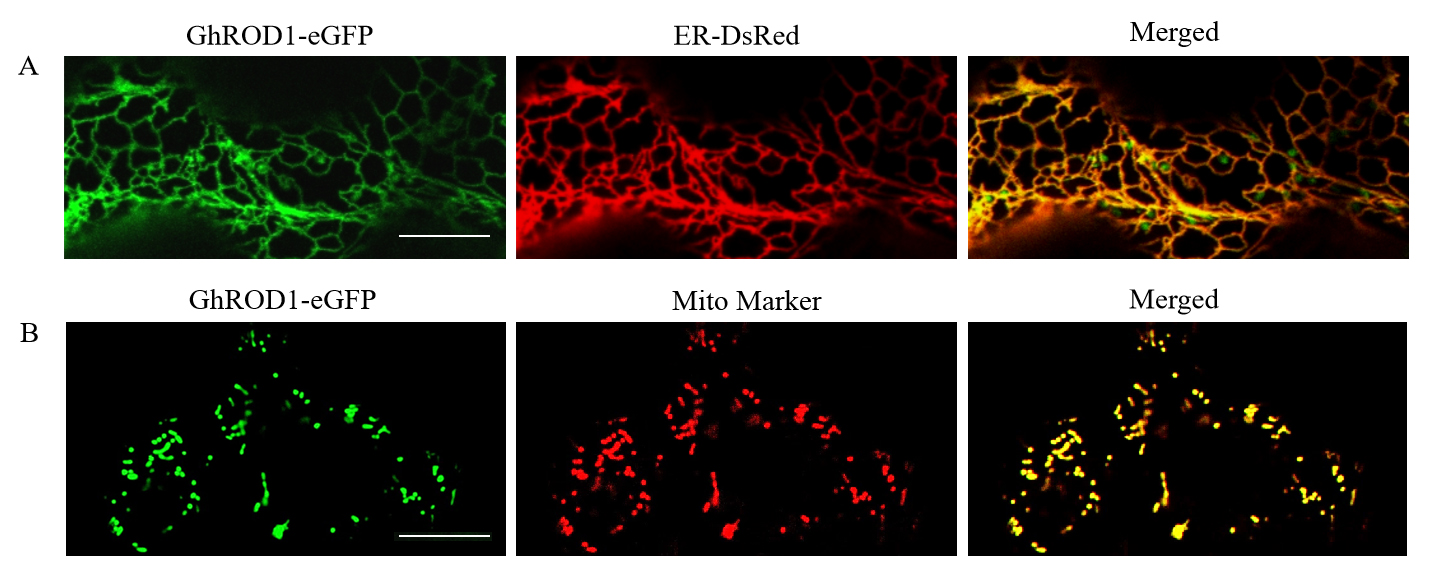

Supplement: Supplementary file 1 [file ijms-25-11242-s001.zip › Figure S4.tif]
